# NO/cGMP/pCREB re-activation reverses cognition deficits and attenuates amyloid-β neuropathology in transgenic models of Alzheimer’s disease

**DOI:** 10.1186/2050-6511-14-S1-P72

**Published:** 2013-08-29

**Authors:** Gregory RJ Thatcher, Jia Luo, Lawren VandeVrede, Zhihui Qin, Sue Lee, Ramy Abdelhamid, Brian M Bennett, Mary Jo LaDu, Leon Tai, John Larson

**Affiliations:** 1 Department of Medicinal Chemistry & Pharmacognosy, University of Illinois College of Pharmacy, University of Illinois at Chicago (UIC), Chicago, IL 60612, USA; 2 UICentre (drug discovery @ UIC), UIC, Chicago, IL 60612, USA; 3 cGC Pharma Inc., Chicago, IL; and Cambridge, MA 02142, USA; 4 Department of Biomedical and Molecular Sciences, Faculty of Health Sciences, Kingston, Ontario, Canada; 5 Department of Anatomy and Cell Biology, UIC, Chicago, IL 60612, USA; 6 Department of Psychiatry, Neuropsychiatric Institute, UIC, Chicago, IL 60612, USA

## Background

An early event in Alzheimer’s Disease (AD) is synaptic failure, making the disease fundamentally a disorder of impaired cognition and memory. Synaptic plasticity requires activation of gene expression programs with dysfunction of the transcription factor cAMP-response element binding protein (CREB) strongly implicated in AD etiology.

## Results and Conclusion

The hypothesis that activation of CREB through NO/cGMP signaling might modify the amyloid-β (Aβ) neuropathology, linked to AD pathogenesis, was demonstrated in both APP/PS1 and 3xTg transgenic mouse models of AD using small molecules, termed nomethiazoles, also designed to provide neuroprotection and attenuate pro-inflammatory cytokine release. Functional restoration of long-term potentiation was shown in hippocampal slices from AD transgenic mice in accord with observation of restoration of cognitive function *in vivo*, and was dependent upon soluble guanylyl cyclase (sGC) activation*.* Levels of pCREB and BDNF were significantly elevated, whereas TNFα, Aβ, oligomeric Aβ_1-42_, and also tau protein were significantly lowered after drug treatment. In the absence of neuronal loss in animal models of AD, neuroprotection was demonstrated in rat primary neurons after oxygen-glucose deprivation or application of oligomeric Aβ. The lead nomethiazole was also studied in a novel transgenic mouse model, E4FAD, which incorporates familial AD mutations, but also has targeted replacement of mouse apolipoprotein-E with human ApoE4, the major genetic risk factor for sporadic and age-related AD.

